# Imaging Atherosclerosis

**DOI:** 10.1161/CIRCRESAHA.115.306247

**Published:** 2016-02-19

**Authors:** Jason M. Tarkin, Marc R. Dweck, Nicholas R. Evans, Richard A.P. Takx, Adam J. Brown, Ahmed Tawakol, Zahi A. Fayad, James H.F. Rudd

**Affiliations:** From the Division of Cardiovascular Medicine, University of Cambridge, Cambridge, UK (J.M.T., A.J.B., J.H.F.R.); Department of Clinical Neuroscience, University of Cambridge, Cambridge, UK (N.R.E.); Centre for Cardiovascular Science, University of Edinburgh, Edinburgh, United Kingdom (M.R.D); Cardiac MR PET CT Program, Massachusetts General Hospital and Harvard Medical School, Boston, MA (R.A.P.T., A.T.); Imaging Sciences Laboratories, Translational and Molecular Imaging Institute, Icahn School of Medicine at Mount Sinai, NY (Z.A.F., M.R.D.); and Department of Cardiology, Zena and Michael A. Wiener Cardiovascular Institute, Icahn School of Medicine at Mount Sinai, NY (Z.A.F.).

**Keywords:** atherosclerosis, coronary artery disease, molecular imaging, multimodal imaging, risk factors

## Abstract

Advances in atherosclerosis imaging technology and research have provided a range of diagnostic tools to characterize high-risk plaque in vivo; however, these important vascular imaging methods additionally promise great scientific and translational applications beyond this quest. When combined with conventional anatomic- and hemodynamic-based assessments of disease severity, cross-sectional multimodal imaging incorporating molecular probes and other novel noninvasive techniques can add detailed interrogation of plaque composition, activity, and overall disease burden. In the catheterization laboratory, intravascular imaging provides unparalleled access to the world beneath the plaque surface, allowing tissue characterization and measurement of cap thickness with micrometer spatial resolution. Atherosclerosis imaging captures key data that reveal snapshots into underlying biology, which can test our understanding of fundamental research questions and shape our approach toward patient management. Imaging can also be used to quantify response to therapeutic interventions and ultimately help predict cardiovascular risk. Although there are undeniable barriers to clinical translation, many of these hold-ups might soon be surpassed by rapidly evolving innovations to improve image acquisition, coregistration, motion correction, and reduce radiation exposure. This article provides a comprehensive review of current and experimental atherosclerosis imaging methods and their uses in research and potential for translation to the clinic.

For many, atherosclerosis occurs as an indolent process arising throughout adult life because of multiple pathological changes triggering lipoprotein dysregulation and immune-cell activation at vulnerable points within the arterial system.^1^ Pathologically, this manifests in a series of histologically and structurally distinct lesion types with varying complexity and propensity to instigate an acute clinical event.^2^ As most individuals with atherosclerosis will never experience overt clinical symptoms related to their disease, it is, therefore, not surprising that >50% of those who die suddenly because of coronary heart disease lack prior warning of their condition.^3^ This tip-of-the-iceberg scenario presents a tremendous diagnostic challenge, underpinning the rising global health burden of cardiovascular disease (CVD). Although preventative strategies and improved treatments adopted mostly by wealthy countries have reduced global age-standardized death rates because of ischemic heart disease and ischemic stroke by 19.5% and 26.6%, respectively, since 1990, these remain the top 2 causes of death worldwide, with increasing prevalence in many lower- and middle-income countries and among the growing aging population.^4^

Well-trodden CVD risk-assessment algorithms and diagnostic pathways have reasonable sensitivity and specificity to identify individuals deemed to be at increased risk of future clinical events, and those with hemodynamically significant intraluminal stenoses that might benefit from a treatment strategy including revascularization. However, of the many available clinical methods used to diagnose atherosclerosis, none can accurately foresee the complex exchange of cellular, molecular, and biomechanical factors heralding sudden symptomatic plaque disruption and its downstream sequelae. Thus, there is a need to focus on emerging atherosclerosis imaging platforms that allow more accurate visualization of culprit pathological processes than standard techniques and to develop these tools to help refine risk assessment models and guide therapy.

## Current Tools and Techniques in the Pipeline

**Figure 1.**
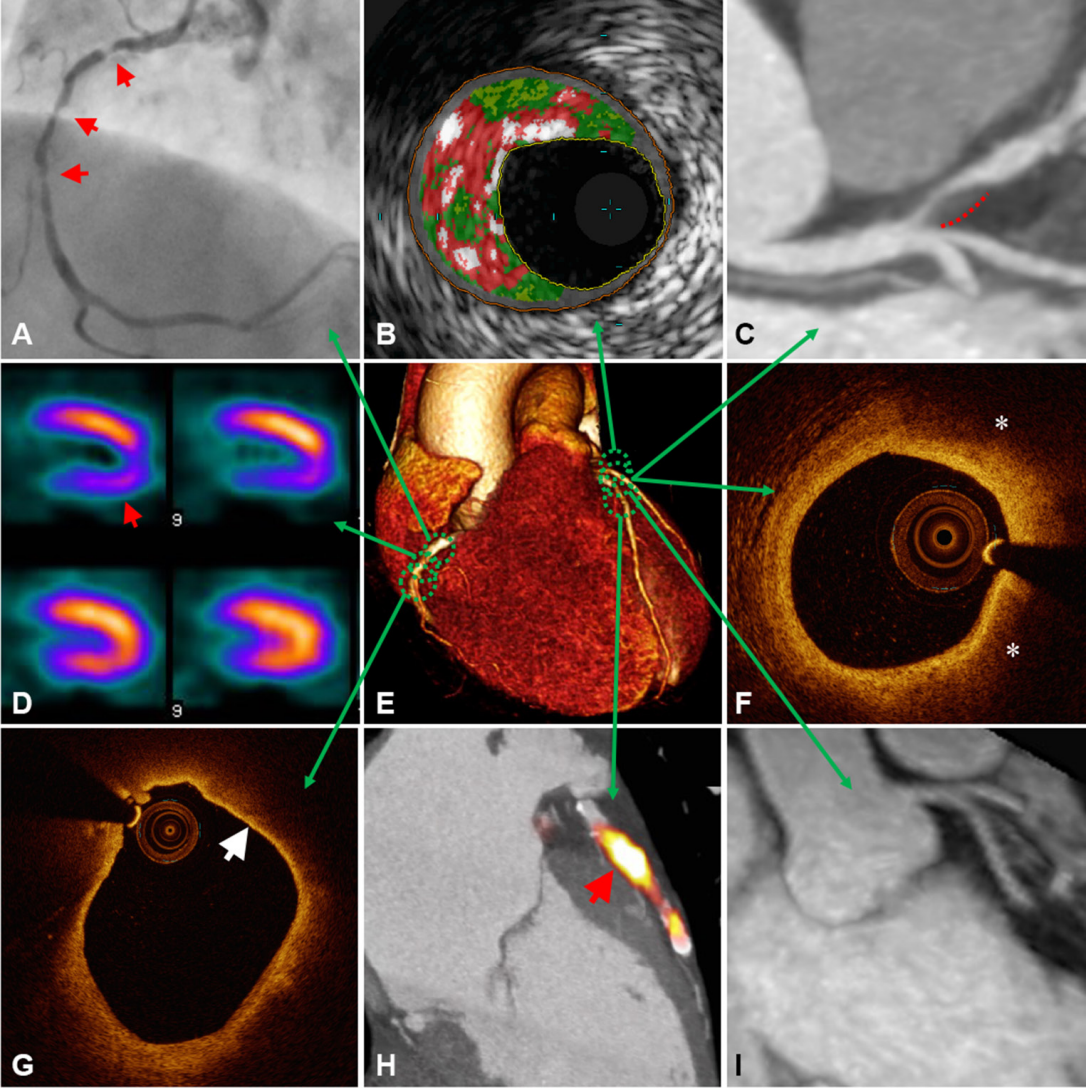
**Multimodal approach to atherosclerosis imaging**. A representative illustration of current and emerging atherosclerosis imaging modalities. Each modality offers unique measurements of disease severity. Together, this information can be used to determine anatomic and hemodynamic consequences of atherosclerosis, complimented by detail on plaque composition, overall disease burden, and current metabolic activity acting within an individual patient. **A**, X-ray angiography showing multiple right coronary artery atherosclerotic lesions (arrows) resulting in significant luminal narrowing; **B**, virtual histology intravascular ultrasound (VH-IVUS) demonstrating coronary plaque with high content of necrotic core (red), as well as dense calcium (white) and fibro-fatty regions (dark/light green); **C**, Computed tomographic (CT) angiography showing noncalcified plaque in the left anterior descending artery with positive remodeling (dashed line); **D**, single-photon emission computed tomography (SPECT) myocardial perfusion scan with stress-induced perfusion defect (arrow); **E**, 3D volume rendered CT whole-heart image; **F**, optical coherence tomography (OCT) image of a coronary plaque showing lipid (*), characterized as signal-poor regions with poorly demarcated borders; **G**, OCT image of a lipid-rich coronary plaque displaying thin overlying fibrous cap (arrow), indicative of thin-cap fibroatheroma; **H**, Fused ^18^F-NaF positron emission tomography (PET)–CT image showing high left anterior descending artery tracer uptake (arrow) revealing active plaque microcalcification; **I**, 3-T magnetic resonance (MR) contrast-angiography performed with dual ECG and respiratory navigator gating showing clear delineation of the proximal left-sided coronary vessels. Panel H adapted from Joshi et al.^5^

Atherosclerosis imaging encompasses an armamentarium of established and experimental radiological methods and modalities. Broadly, these techniques can be used to detect anatomic and physiological consequences of long-standing atherosclerosis, to provide detail on plaque composition and molecular activity, and to estimate biomechanical stresses acting within the arterial system (Figure 1). Together, these methods provide measures of disease severity, which are indispensable to everyday clinical practice and cardiovascular research.

### Anatomic Imaging

Contrast luminography is the gold-standard anatomic atherosclerosis imaging technique. For diagnosis of coronary artery disease (CAD), invasive angiography is most often performed; this involves selective intubation of the coronary ostia with a preshaped catheter introduced via a peripheral arterial sheath, used to inject radio-opaque contrast under x-ray fluoroscopy. The high-diagnostic accuracy afforded by superior spatial (0.1–0.2 mm) and temporal (10 ms) resolution is unmatched by noninvasive techniques. Hence, invasive angiography remains the best anatomic reference test to determine the severity of coronary luminal obstruction and is commonly used to guide clinical management, particularly, when contemplating revascularization. However, the overall low diagnostic yield of elective coronary angiography supports initial noninvasive testing and a gate-keeper approach.^6,7^ Noninvasive angiography is possible when using coronary computed tomographic angiography (CCTA) or magnetic resonance angiography (MRA) combined with ECG-gating to account for cardiac movement. CCTA is preferred over MRA for anatomic coronary imaging, although MRA is commonly used to evaluate anomalous coronary vessels in children or young women to avoid radiation exposure. MRA is best-suited for imaging large, static vessels such as the carotid arteries, peripheral arteries or aorta.

#### Coronary Computed Tomographic Angiography

Multidetector CCTA is a useful first-line diagnostic test for appropriately selected patients with symptoms of angina and a low-to-moderate pretest probability of CAD. In this context, CCTA has excellent negative predictive value, providing a reliable means to exclude CAD when the clinical diagnosis is doubtful.^8^ Although precise quantification of anatomic stenosis severity in patients with established CAD can be challenging owing to image artefacts created by coronary calcification, CCTA is nonetheless an accurate test with good sensitivity to detect anatomically significant CAD when compared with invasive angiography in large, prospective, multicenter trials.^9,10^ CCTA is often performed in conjunction with coronary artery calcification (CAC) imaging, a risk stratification tool that gives an overall estimate of disease burden and risk of future events.^11^

Current evidence does not support the use of CCTA over clinical risk±CAC scores in asymptomatic patients^12^; however, CCTA does add significant diagnostic benefit when added to standard care for patients with suspected angina and might lead to changes in management that improve long-term survival in this group.^13^ It has also been shown to be superior to noninvasive functional testing for detection of angiographically significant coronary stenosis in patients with CAC <400.^14,15^ When there is a high pretest probability of CAD or raised CAC score, diagnostic x-ray angiography is often preferred first-line over CCTA and other noninvasive tests. Among the growing list of other clinical indications for anatomic CCTA assessment are to improve early clinical decision making for patients with acute chest pain in the emergency department, as an adjunct to invasive angiography when planning complex percutaneous intervention, for detection of in-stent restenosis; to check patency of coronary artery bypass grafts; to exclude CAD as a cause of left ventricular dysfunction, or before cardiac valve surgery; and to understand the 3D anatomic relationship of an anomalous coronary artery to its surrounding structures.^16,17^

The new generations of computed tomography (CT) scanners with larger detector arrays, more detector rows, and dual-source systems offer high-quality CCTA images with less noise, less artifact, and at lower radiation doses compared with earlier models. The spatial resolution of CCTA in the clinical setting is roughly 0.5 to 0.6 mm. Depending on the gantry rotation time, and whether a dual-source scanner or single-heart beat acquisition is used, the temporal resolution varies from 66 to 210 ms. Because of improved temporal resolution, good quality images can even be obtained using prospective ECG-triggering in some patients with an elevated heart rate or arrhythmia, resulting in significantly reduced radiation exposure compared with retrospective scanning.^18^ Diagnostic accuracy of CCTA when performed in patients with high CAC might also be enhanced by using calcium subtraction methods.^19^

#### Magnetic Resonance Angiography

Although 2D-Doppler ultrasound is typically the most accessible first-line test to assess for carotid artery disease in patients with recent stroke or transient ischemic attack, MRA is also used routinely for this purpose. Compared with CT, MRI has superior soft-tissue characterization and lacks ionizing radiation. However, in the coronary circulation, obtaining good-quality MRA images is challenging because of motion artefacts arising during prolonged acquisition time and difficulties achieving satisfactory contrast-to-noise ratio, spatial resolution, and volumetric coverage. It is, therefore, not currently recommended for routine clinical use. Despite these challenges, coronary MRA is a rapidly developing modality, and with recent technological improvements, can provide reliable imaging of the proximal and midvessels. These advances include free-breathing 3D whole-heart acquisition with ECG-gating and navigator respiratory motion correction, high-field magnetic resonance (MR), 32-channel coils with high parallel imaging to accelerate acquisition, T1/T2 spin preparations, and different pulsed sequences to increase signal/contrast-to-noise ratios.^20,21^ When using noncontrast bright-blood techniques for coronary MRA, which rely on the high T2/T1 ratio of blood to act as an intrinsic contrast agent, the need for potentially nephrotoxic contrast agents can be avoided.

At 1.5 T, whole-heart MRA can identify coronary stenoses >50% with reasonable certainty^22,23^ and identify left main stem and three-vessel CAD in 94%.^24^ Moreover, significantly improved diagnostic accuracy has been reported with 3T contrast-enhanced whole-heart MRA when compared with x-ray angiography in a single center.^25^ Absence of significant stenosis on coronary MRA has also been associated with a low risk of subsequent cardiac events when monitored over 2 years.^26^ In the future, when scanning patients with suspected CAD, integration of coronary MRA with other MR functionalities could potentially provide a comprehensive evaluation of coronary anatomy, ventricular wall motion, myocardial perfusion, viability, and scarring.

### Functional Imaging

For patients with persistent angina despite medical therapy, a diagnostic strategy that incorporates functional testing over anatomic assessment alone results in overall better symptom control and less invasive procedures.^27^ This is in part due to the fact that percentage luminal stenosis does not reliably correlate with hemodynamic obstruction or ischemic burden. However, while detection of flow-limiting CAD resulting in myocardial ischemia relates an adverse prognosis, studies evaluating the effects of ischemia reduction after percutaneous coronary intervention (PCI) for stable angina have consistently failed to show significant reduction in myocardial infarction (MI) or mortality. This finding indicates that functional stenosis severity might serve as a marker of overall disease burden rather than the direct cause of most future clinical events. Diffuse atherosclerosis with associated microcirculatory dysfunction may additionally contribute to increased risk.^28^

Hemodynamically significant CAD can be determined noninvasively with stress imaging or by pressure-wire assessment during invasive angiography, although these tests are often underused before elective PCI.^29^ For functional assessment, exercise is either performed before imaging or simulated pharmacologically with adenosine or another stress-inducing agent. Noninvasive functional imaging modalities include stress echocardiography, cardiac MR with stress perfusion, and nuclear myocardial perfusion scanning with single photon energy computed tomography (SPECT) or positron emission tomography (PET); ^18^F-flurpiridaz is a promising novel PET perfusion tracer currently being evaluated in phase 3 clinical trials.^30^ When comparing these methods, there are advantages and limitations to each, but they are generally equally effective and selection is typically guided by local experience and availability. Fractional flow reserve (FFR) is the most popular invasive functional method, which provides a reliable pressure-based marker of relative coronary flow reserve obtained during maximum pharmacological hyperemia, which is comparable with absolute myocardial blood flow measured by quantitative PET. However, in 1 study, >40% of vessels reported as FFR positive (<0.80) had normal or only mildly reduced coronary flow capacity indicating that significant myocardial ischemia was unlikely in these patients.^31^ CT perfusion is another emerging technique, which if combined with CCTA has reasonable accuracy when evaluated against SPECT, FFR, and MR perfusion.^32–34^ First pass MRI perfusion imaging might also be feasible using dynamic nuclear polarization with compounds, such as ^13^C urea, which has the potential to increase signal-to-noise ratio by >10 000-fold compared with thermally polarized spins.^35^

### Imaging Plaque Morphology and Composition

Beyond conventional anatomic and hemodynamic assessments of lesion severity, detailed plaque characterization can be obtained when imaging the vessel wall. Data from autopsy studies performed in patients with CAD who died suddenly provide the histopathologic basis for high-risk plaque identification and have paved the way for so-called vulnerable plaque imaging.^36^ From this work, we know that the most common underlying plaque morphology leading to MI comprises a thin ruptured fibrous cap with heavy macrophage infiltration and few smooth muscle cells, large necrotic core and overlying intraluminal thrombosis. Intimal neovascularization is a source of intraplaque hemorrhage, which contributes to increased risk of plaque rupture.^37^ Plaque erosion occurs in 30% to 35% of sudden coronary death cases, and thrombi attributed to calcified nodules in 2% to 7%.^38^

The thin-cap (<65 μm) fibroatheroma (TCFA) bears close resemblance to ruptured plaque but lacks luminal thrombosis and is, therefore, regarded as the most likely precursor lesion to plaque rupture. Indeed, cap thickness is the best histological discriminator of coronary plaque type, followed by macrophage infiltration and necrotic core.^39^ The histopathologic appearance of recently symptomatic carotid plaques is similar to culprit coronary lesions,^40^ although cap thickness seems less important.^41^ As most rupture-prone coronary plaques occur in a limited, focal distribution, clustered mainly within the proximal coronary vasculature,^42^ both invasive and noninvasive plaque characterization is feasible. Thin-cap, large necrotic core, positive remodeling, microcalcification, and neovascularization are among the list of recognized high-risk coronary plaque features detectable in vivo; however, the role of high-risk plaque imaging in routine clinical practice has yet to be determined.

#### Intravascular Coronary Imaging

Intravascular coronary imaging with ultrasound (IVUS), optical coherence tomography (OCT), and near infrared-spectroscopy (NIRS) can provide detailed information about coronary plaque composition in patients undergoing invasive angiography. An IVUS catheter, constructed from either an electronic phased array or single-element design, generates sound waves in the 20 to 60 MHz range because of high-speed oscillatory movement of a piezoelectronic transducer. Gray-scale IVUS has limited ability to differentiate individual plaque components, but spectral analysis of the backscattered radiofrequency data with, for example, virtual histology (VH)-IVUS can be used to detect necrotic core, dense calcium, fibrous, and fibrofatty plaque with reasonable accuracy.^43^ However, because of increased noise and artifacts, image interpretation can be difficult; furthermore, IVUS has insufficient spatial resolution to reliably and reproducibly detect thin fibrous cap.^44^

OCT uses near infrared light (1.3 μm wavelength) emitted through a fiberoptic wire with rotating lens to achieve exceptionally high spatial resolution (10–15 μm), providing accurate measurement of fibrous cap thickness with strong correlation to histology,^45^ and good sensitivity and specificity to distinguish plaque type.^46^ However, correct differentiation between calcium and lipid pool can be challenging with OCT, and its limited tissue penetration (1–3 mm) makes assessment of the entire plaque volume impossible. Image acquisition also requires a blood-free field, achieved through the injection of saline or contrast flushing during pullback. Other potential applications of OCT include elastography, OCT Doppler, and polarization-sensitive OCT.^47^

Because of its superior resolution, certain OCT appearances might provide a glimpse into variations in plaque composition at the cellular level. In an ex vivo study using raw uncompressed data, OCT was used to quantify macrophages within the fibrous cap, seen as bright spots with higher signal intensity than surrounding structures and a sudden drop off in OCT signal.^48^ Although an intriguing finding, further work has revealed that only 23% of bright-spot positive regions on OCT specifically represent macrophages alone.^49^ Another ex vivo study in human coronaries using micro-OCT (spatial resolution of 1 μm) has revealed striking images of cellular and subcellular structures, including leukocytes tethered to endothelial surface in similar appearance to electron microscopy.^50^ For identification of TCFA, VH-IVUS and OCT have similar diagnostic accuracies (76–79%), which might in future be improved by introduction of hybrid imaging catheters.^51^

NIRS uses diffuse reflectance near-infrared light (0.8–2.5 μm wavelength) to create a chemogram of vessel wall components, based on detection of varied absorption and scattering patterns.^52^ Although NIRS can identify lipid content underlying high-risk plaque in human arteries through blood, as shown by ex vivo work,^53^ and clinical imaging,^54^ its major limitation is that it does not provide any structural information on the plaque. This limitation can, in part, be overcome by NIRS–IVUS hybrid imaging, although this hybrid catheter still cannot robustly assess cap thickness.^55^

#### CT-Derived Plaque Morphology

In addition to defining coronary anatomy and luminal stenosis severity, CCTA can provide information on plaque morphology and composition. Cap thickness and necrotic core are among the most important histological predictors of plaque rupture.^38^ Although the spatial resolution of CCTA is insufficient to allow measurement of cap thickness, necrotic cores of TCFAs are typically large enough for detection by CT.^56^ Plaques can be readily classified as calcified, partially calcified (<50%), or noncalcified plaques using CCTA. When assessing plaque volume, CCTA tends to underestimate the size of noncalcified plaques and overestimate calcified plaque because of blooming artifact.^57^ The sensitivity of CCTA to detect noncalcific plaques with >1 mm intimal thickness on IVUS is roughly 90%.^58^

When compared with VH-IVUS, fibrous plaques display high attenuation on CT, whereas low attenuation occurs in relation to necrotic core and fibrofatty tissue.^59^ Good correlations have also been shown between high-risk CT features and TCFA on OCT,^60^ and CT-derived plaque burden to cholesterol deposition on NIRS.^61^ On the basis of IVUS studies, Hounsfield unit <30 on CCTA has been proposed as a cutoff for identification of lipid rich plaque, with 30 to 150 Hounsfield unit for fibrous and >220 Hounsfield unit calcific.^62^ However, using absolute CT attenuation values to determine plaque composition is challenging because of the influence of various factors, including size of necrotic core, wall thickness, measurement point, density of intraluminal contrast, slice thickness, and reconstruction filter.^63,64^ Contrast-adjusted attenuation ranges can potentially improve accuracy of CCTA plaque component analysis.^65^

**Figure 2.**
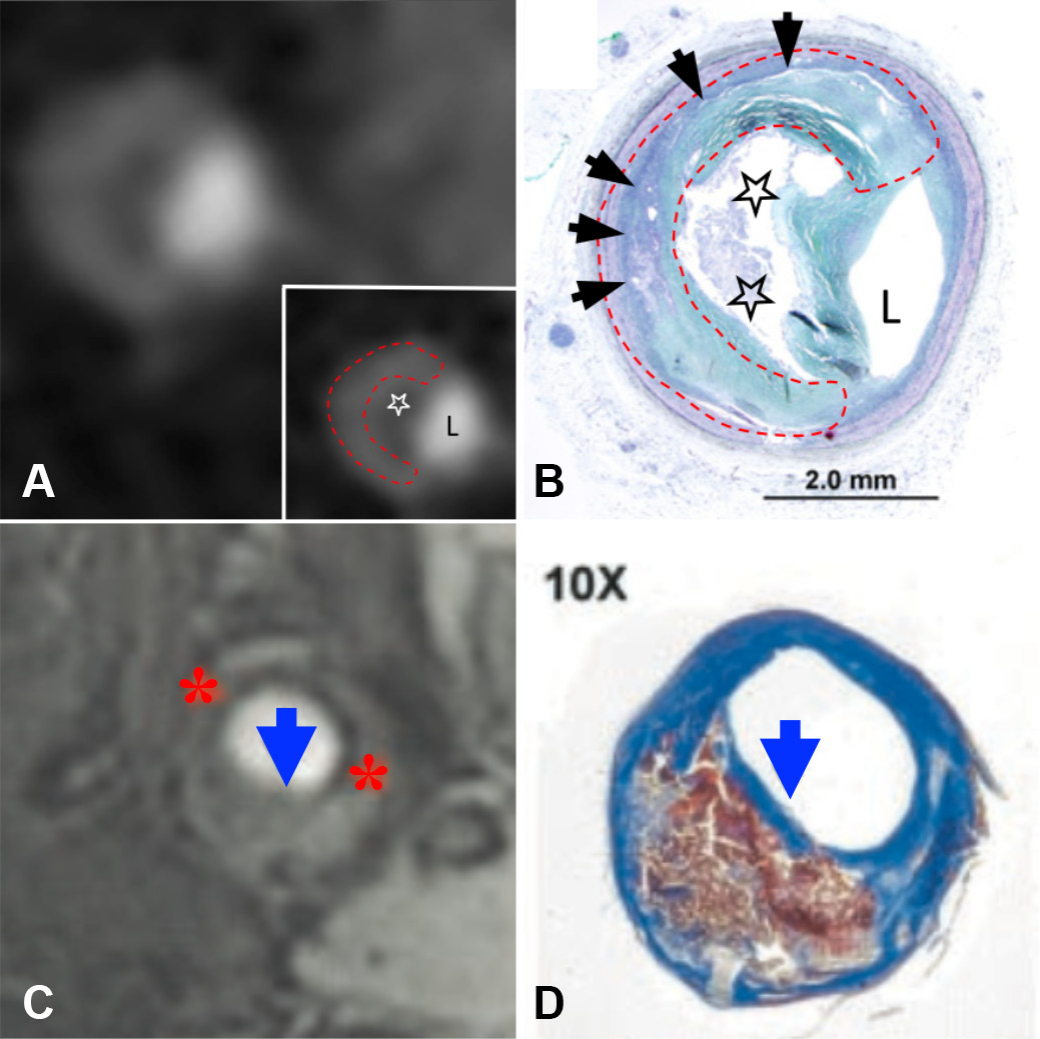
**Noninvasive imaging of atherosclerotic plaque composition**. Coronary artery shown in cross section, demonstrating napkin-ring sign on contrast-enhanced computed tomography (CT) (**A**) with corresponding histology confirming presence of advanced fibroatheroma (**B**). Area of low attenuation (white star) abutting lumen (L) seen on CT corresponds to necrotic core (black stars) on histology. High-attenuation circumferential outer rim on CT image (dashed red line) corresponds to fibrous plaque tissue (black arrows). Magnetic resonance image (MRI) of carotid artery shown in axial view with 3D time-of-flight MRI (**C**). Thin fibrous cap (blue arrows) demonstrated by missing area of hypodense juxtaluminal band (red asterisk) on MRI, confirmed by histology (**D**). Adapted from Maurovich-Horvat et al^70^ (**A** and **B**; copyright ©2010, Elsevier) and Yuan et al^74^ (**C** and **D**; copyright ©2002, American Heart Association, Inc) with permission of the publishers.

Unstable lesions imaged with CCTA in patients with acute coronary syndrome (ACS) tend be noncalcified, with low attenuation and spotty calcification, larger plaque volume, and higher remodeling index compared with stable lesions in patients with chronic stable angina.^62,66^ Positive (outward) remodeling occurs because of compensatory enlargement of the vessel wall, leading to high-volume plaque with often little luminal narrowing; a feature associated with large lipid core and high macrophage count.^67^ The threshold for positive remodeling on CT is cross-sectional area >10% of the adjacent reference segment, and spotty calcification is defined as <3 mm in all directions.^62^ Spotty calcification reflects small calcific deposits within the plaque architecture, rather than true microcalcification, which occurs in response to inflammation and acts to destabilize the plaque by influencing local stress concentration.^68^ When detected by IVUS, spotty calcification is associated with diffuse atherosclerosis and accelerated disease progression.^69^ The CT napkin-ring sign demonstrates an area of low attenuation adjacent to the vessel lumen, with surrounding higher-attenuation ring (Figure 2). This sign is suggestive of lipid-rich necrotic core and fibrous components of TCFA.^70^ Low attenuation plaque, positive remodeling, and napkin-ring sign on CT are prognostic indicators linked to increased risk of MI.^71,72^ Interestingly, these high-risk plaque features are also 3 to 5 times more likely to occur in relation to FFR-positive lesions than in nonobstructive disease.^73^

#### MR-Derived Plaque Morphology

**Figure 3.**
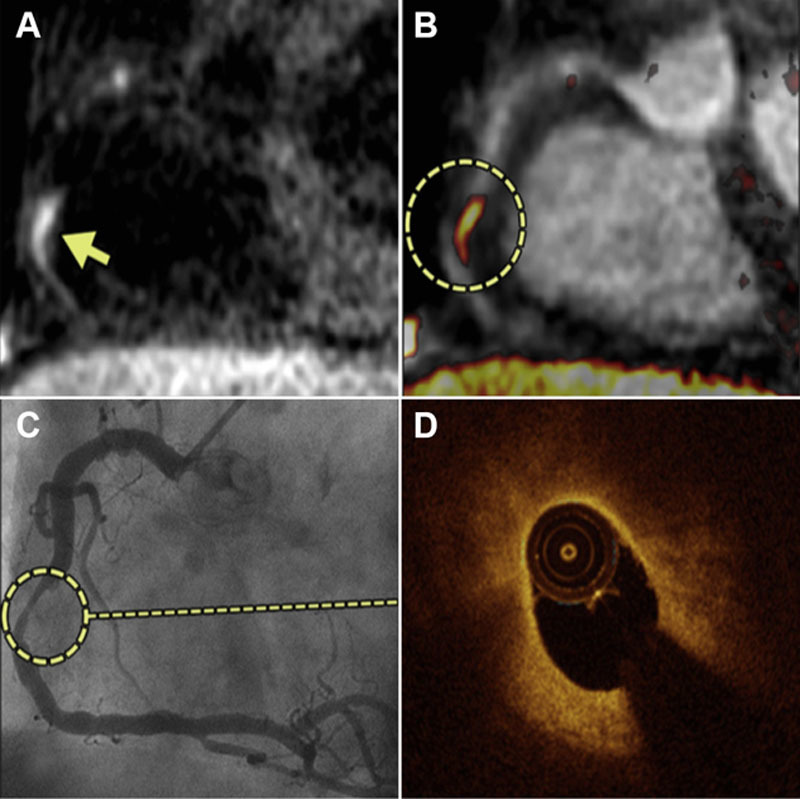
**Hyperintense coronary signal on T1-weighted magnetic resonance image (MRI**). **A**, T1-weighted MRI showing hyperintense signal within the wall of the right coronary artery (arrow), highlighted by fused MRI (circle, **B**); **C**, X-ray angiography shows severe stenosis in corresponding coronary segment, with lipid-rich plaque in this same region on optical coherence tomography (OCT) (**D**). Adapted from Matsumoto et al^78^ with permission of the publisher. Copyright ©2015, Elsevier.

Coronary plaque analysis with MRI is much less useful than CCTA for clinical purposes; however, wall thickness can be measured with this method in proximal vessels.^75^ Using black-blood MRI, positive remodeling and increased coronary wall thickness has been shown in asymptomatic patients with cardiovascular risk factors.^76^ Visualization of wall edema relating to culprit ACS lesions using T2-weighted short inversion recovery sequence MRI has also been reported.^77^ Furthermore, hyperintense coronary signal on T1-weighed MRI might serve as a marker of high-risk plaque, which has been linked to clinical angina severity and increased cardiovascular risk (Figure 3).^78,79^ Hyperintense T1 plaque signal is most likely because of methemoglobin formation during subclinical plaque rupture or plaque hemorrhage; this is the most promising current MR technique for identifying high-risk coronary plaques.

Unlike coronary imaging, carotid arteries are relatively stationary and of sufficient caliber to allow interrogation of plaque morphology using multicontrast weighted MRI or gadolinium-based intravenous contrast. T1 and T2 sequences on multicontrast weighted MRI distinguish plaque components, which exhibit differing relaxation properties and signal intensity. Using this technique, carotid wall thickness can be accurately measured without intravenous contrast on a standard 1.5-T scanner, although better image quality due to improved signal- and contrast-to-noise ratios is seen with higher-field strength 3.0-T MRI.^80^ Fibrous tissue displays low signal on T1 and high-signal on T2-weighted MRI, whereas calcium is hypodense on both. With high-resolution 3D time-of-flight MRI, fibrous cap appears as a juxtaluminal band of low signal, which is absent in the presence of a thin or ruptured cap (Figure 2). Cap rupture detected by MRI has been shown in relation to recently symptomatic carotid plaques.^74^ Intraplaque hemorrhage causing high-intensity T1-signal in carotids arteries^81^ has been correlated to increased C-reactive protein,^82^ symptomatic cap rupture,^83^ and increased risk of future stroke.^84^

MRI with gadolinium-based contrast can be used to quantify the dimensions of fibrous cap and lipid-rich necrotic core.^85^ Lipid core displays lower enhancement than surrounding fibrous tissue on T1-weighted images post intravenous contrast. In addition to thin, or ruptured, cap and intraplaque hemorrhage, increased lipid core on MRI is an important prognostic marker.^86^ Dynamic contrast enhancement has also been applied to MR carotid imaging. With this method, dynamic images acquired pre- and post-gadolinium contrast injection are analyzed using kinetic modeling to derive transfer constant (K^trans^), which is linked to carotid plaque macrophage content and neovascularization.^87^

### Molecular Imaging

Mechanisms underlying the pathobiology of atherosclerosis and its clinical consequences can be illuminated using highly sensitive molecular imaging probes. Noninvasive nuclear and MR-based molecular imaging platforms have been most extensively studied in vivo, although molecular CT, ultrasound, and near-infrared fluorescence (NIRF) are among other emerging research methods. The advantage of PET over other techniques, including SPECT and MRI, is its superior sensitivity to detect molecular signals, even at picomolar tissue concentrations. However, limited spatial resolution (4–5 mm) means that images must be coregistered with CT or MRI for precise anatomic localization of the PET signal. Among other applications, molecular probes used in atherosclerosis imaging can be used to quantify vascular inflammation, early calcification, plaque hypoxia, and neoangiogenesis.

#### Molecular Methods for Imaging Inflammation

Within the arterial wall, innate and adaptive immune responses triggered largely by clinical cardiovascular risk factors are major determinants of atherosclerotic progression and plaque rupture. Macrophages direct proinflammatory cell signaling cascades underlying high-risk plaque morphology, thus, presenting an attractive molecular imaging target to track vascular inflammation.

### Nuclear Inflammation Imaging: PET and SPECT

^18^F-fluorodeoxyglucose (FDG) is a radio-labeled glucose analogue used commonly in PET imaging for a variety of diagnostic purposes. Its half-life is 110 minutes and maximum positron range 2.4 mm. After intravenous injection, decay results in the emission of positrons and occurs at a predictable rate. ^18^F-FDG is taken up by cells that metabolize glucose, where it becomes metabolically trapped after phosphorylation into ^18^F-FDG-6-phosphate, as it lacks the necessary 2′ hydroxyl group to continue glycolysis. The PET scanner detects annihilation (γ) photons, which result when an emitted positron encounters an electron. Tomographic images displaying the distribution of injected radio-labeled tracer within the body are reconstructed in 3D, with corrections applied for attenuation, dead time, scatter, and random coincidences. Intracellular accumulation of ^18^F-FDG can be used as a biomarker of metabolic activity. In atherosclerosis, vascular ^18^F-FDG uptake at late time points reflects increased activity of macrophages and to a lesser extent other immune cell types. For the purpose of vascular imaging, the optimal dose is 185 to 300 MBq and circulation times of over 2 hours are recommended.^88^

**Figure 4.**
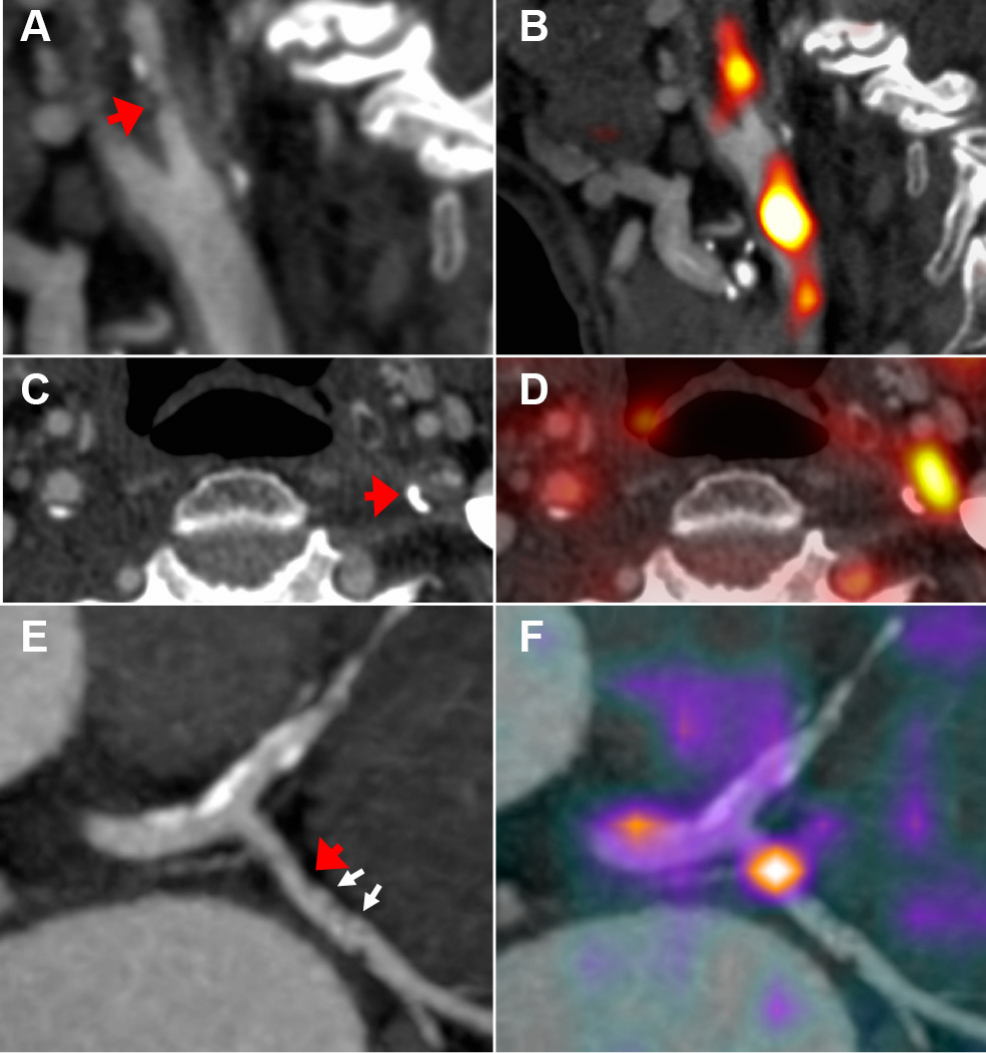
**Positron emission tomography (PET) inflammation imaging**. Computed tomographic (CT) angiography of symptomatic left internal carotid stenosis (arrows); sagittal (**A**) and axial (**C**) views. **B, D**, Fused ^18^F-fluorodeoxyglucose (FDG) PET–CT demonstrates high uptake relating to the symptomatic carotid plaque. **E**, Coronary CT angiogram of left circumflex coronary artery lesion (red arrow) with spotty calcification (white arrows); **F**, fused ^68^Ga-DOTATATE PET–CT demonstrates high signal in relation to the inflamed coronary plaque.

^18^F-FDG PET is an important research tool for studying vascular inflammation (Figure 4), which has been validated by both clinical and preclinical studies across several vascular territories. The ^18^F-FDG signal shows strong correlation to macrophage density (%CD68 staining) within excised carotid plaques, presence of cardiovascular risk factors, Framingham risk score, and inflammatory biomarkers.^89^ Carotid artery ^18^F-FDG uptake also relates to high-risk plaque morphology,^90^ and carotid and aortic signals can help predict future cardiovascular risk.^91–93^ In atherosclerosis, ^18^F-FDG uptake seems to be highest during early foam cell formation^94^ and does not typically colocalize with areas of macroscopic vascular calcification.^95^ Interestingly, ^18^F-FDG signals are higher in low shear-stress induced macrophage-rich carotid plaques compared with stable plaques in mice,^96^ and possibly also among proinflammatory M1 macrophage subtypes.^97^ Although measurement of vascular ^18^F-FDG signals using established techniques is highly reproducible with low short-term interscan variability, accuracy of this method could be further improved by adoption of standardized imaging protocols.^98^

However, as ^18^F-FDG is taken up by all cells with active glucose metabolism, it provides a nonspecific marker of inflammation in atherosclerosis. Vascular ^18^F-FDG uptake is also influenced by plaque hypoxia^99^ and the efficiency of tracer delivery by the microcirculation.^100^ Moreover, high myocardial muscle cell uptake often prevents interpretation of coronary signals, even with strict dietary manipulation.^101^ As a result of these limitations, other PET tracers have been evaluated for use in atherosclerosis imaging. Several of these tracers might be specific for macrophage activity, and better suited for coronary imaging than ^18^F-FDG because of low myocardial uptake, including ^11^C-PK11195 and other TSPO (translocator protein) receptor ligands, ^18^C-choline, and somatostatin receptor binding tracers, such as ^68^Ga-DOTATATE ([1,4,7,10-tetraazacyclododecane-N,N',N'',N'''-tetraacetic acid]-D-Phe(1),Tyr(3)-octreotate).^89^ Macrophage and hematopoietic cell proliferation have also been imaged using ^18^F-fluorothymidine PET in preclinical atherosclerosis models and humans; in this study, higher carotid and aortic ^18^F-fluorothymidine uptake was observed in patients with CVD risk factors compared with controls, as well as a weak positive correlation between ^18^F-fluorothymidine and ^18^F-FDG aortic uptake.^102^

Several SPECT tracers have also been evaluated for inflammation imaging. Radio-isotopes used to make SPECT tracers typically have longer half-lives and are more widely accessible than those used for PET. Although SPECT has a lower spatial resolution (10–16 mm) compared with PET, image quality can potentially be improved by use of new scanners equipped with cadmium–zinc–telluride solid-state detectors.^103^ Among the list of experimental targets for SPECT inflammation imaging are IL-2 receptors on activated T lymphocytes,^104^ folate receptor-β expressed by M2 macrophages,^105^ endothelial vascular cell adhesion molecule (VCAM-1) expression,^106^ and exposed phosphatidylserine on cell surface of apoptotic macrophages.^107^

### Nanoparticle Imaging: MRI, CT, and Ultrasound

Nanoparticles used in molecular atherosclerosis imaging are typically 10 to 100 nm in size. Paramagnetic iron oxide particles, such as ultrasmall supraparamagnetic iron oxide (USPIO), generate MRI signal drop when internalized by macrophages owing to changes in the local magnetic field causing T2-shortening.^21,108^ USPIO signal drop has been detected in patients with symptomatic carotid plaques^109^ and correlates to macrophage-rich plaque areas.^110^ Experimental conjugated iron oxide particles have been developed to target P-selectin and VCAM-1 adhesion molecules,^111^ and scavenger receptors on foam cells.^112^ Plaque imaging using an elastin-based MRI contrast agent has also been performed in preclinical studies.^113^

N1177 is a suspension of crystalline iodinated nanoparticles with high affinity for macrophages, which has been imaged using CT in a rabbit model.^114^ A gold high-density lipoprotein nanoparticle has also been tested in a preclinical atherosclerosis model, using spectral multicolor CT to identify the gold contrast agent based on its energy-dependant photon attenuation.^115^ Contrast-enhanced ultrasound using targeted microbubbles is another molecular imaging technique. Potential advantages of ultrasound over other methods include portability, low equipment cost, and speed of acquisition. Microbubbles contain compressed gas, which undergo volumetric oscillation and expansion in an acoustic field.^116^ Signal from targeted microbubbles retained in tissue can be destroyed with low-frequency, high amplitude ultrasound energy creating a null signal after image acquisition allowing administration of subsequent target agents. In preclinical studies, microbubbles conjugated to activated neutrophils, α_5_-integrins, VCAM-1,^117^ and P-selectin have been tested.^118^

### Near-Infrared Fluorescence

Intravascular NIRF is an emerging molecular imaging method that lacks ionizing radiation. In a feasibility study, a 2D rotational 2.9-F automated pullback intravascular NIRF catheter with protease-activated fluorescence agent was successfully used to obtain 360° high-resolution real-time images of arterial inflammation in a rabbit atherosclerosis model and after stent implantation.^119^ A fully integrated high-speed NIRF-OCT imaging catheter has also been tested, using indocyanine green to detect inflamed lipid-rich atheroma.^120^ Indocyanine green is currently used for clinical NIRF ophthalmologic imaging. Translation of NIRF technology to clinical atherosclerosis imaging trials is expected within the next 1 to 2 years.^121^

### PET Imaging of Microcalcification, Hypoxia, and Neoangiogenesis

Aside from inflammation, several other molecular processes that contribute to the pathogenesis of atherosclerosis can be imaged using PET. Early vascular calcification occurring in response to intense plaque inflammation, and below the resolution of CT, can be detected using ^18^F-sodium fluoride (NaF) in advanced high-risk stable and culprit coronary lesions,^5^ and possibly also in earlier stages during neointimal thickening.^122^ In symptomatic carotid arteries, ^18^F-NaF binding takes place in areas of pathological mineralization and is related to surface area of exposed hydroxyapatite.^123^ Hypoxia develops deep within advanced atheroma, where it acts as a stimulus for new microvessel formation and promotes low-density lipoprotein accumulation and inflammation.^124^ Using ^18^F-HX4 (flortanidazole), plaque hypoxia has been shown in relation to carotid wall thickness and ^18^F-FDG uptake.^125^ ^18^F-fluoromisonidazole has also been applied to study atherosclerosis and correlated to histologically determined hypoxic plaque areas in preclinical and clinical studies.^126,127^ By targeting integrin ανβ3 expression in angiogenic endothelial cells, neoangiogenesis can also potentially be imaged using R-G-D-based tracers with PET^128^ and SPECT.^129^

### Biomechanical Analysis

The nonuniform distribution of atherosclerotic lesions within the vascular system suggests local stimuli contribute to plaque initiation and growth. Specific points within the arterial system seem to be preferentially affected, including bifurcations and the inner curvature of vessels, regions where wall shear stress is typically reduced. Shear stresses result from blood flow creating frictional (axial) forces on the endothelial surface and changes in shear stress induced by arterial geometry are known to result in endothelial injury, inflammation, and altered gene expression influencing vasoreactivity and vessel remodeling.^130^ Persistently low shear-stress seems to be the most important biomechanical factor for TCFA formation.^131^ Structural stress because of tensile (circumferential) forces may also regulate plaque behavior, dictating its ability to withstand mechanical load.^132^ The influence of structural stress in plaques is most important in the presence of a weakened cap, where high mechanical stress is most likely to promote plaque rupture. Computational modeling of shear and structural stresses is possible using data from noninvasive and invasive imaging.

**Figure 5.**
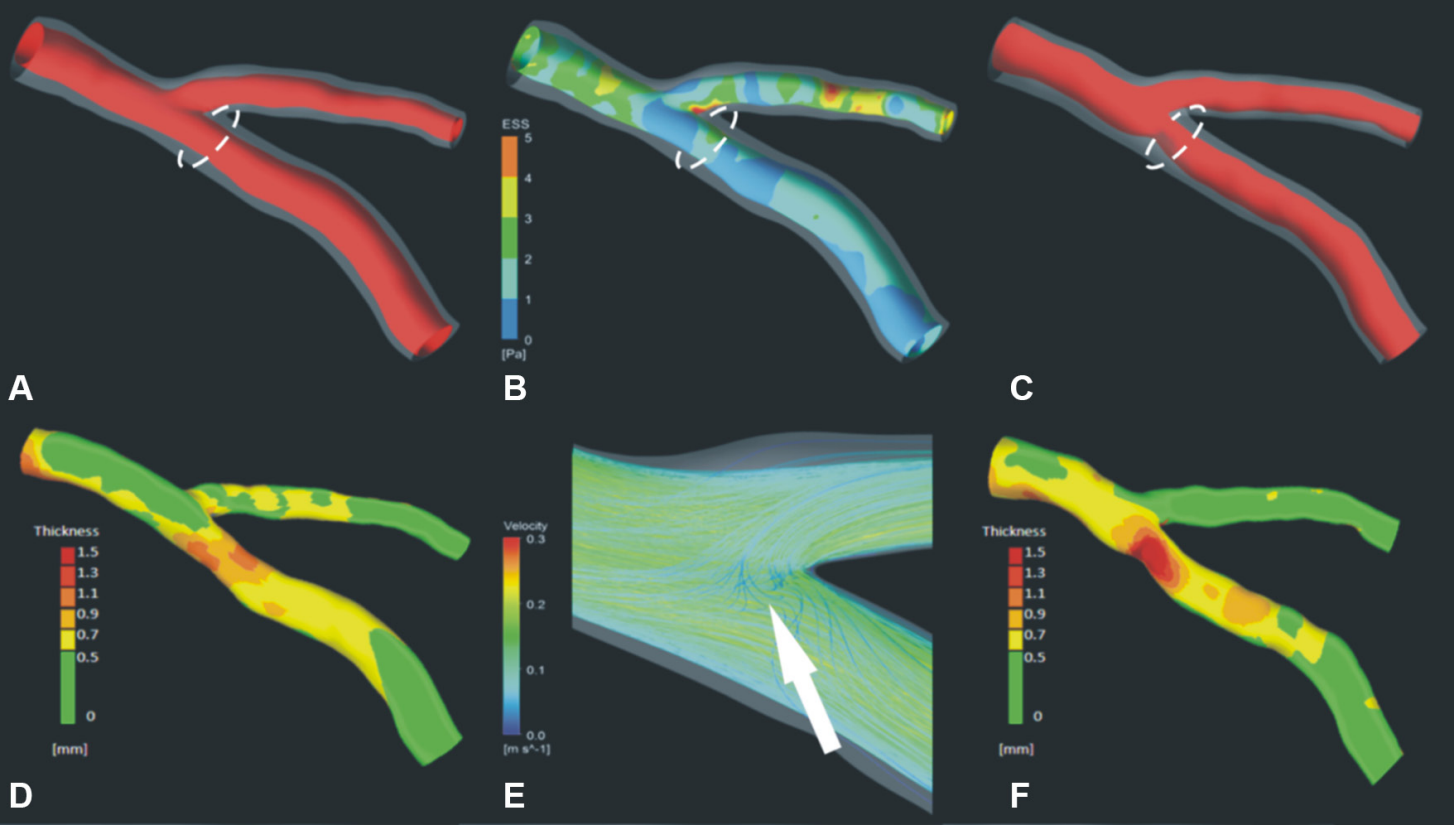
**Shear stress simulation and plaque progression**. 3D coronary reconstruction at baseline (**A**) and 3-year follow-up (**C**); outer vessel-wall shown in a semitransparent fashion to allow visualization of plaque distribution. **B, E**, Shear stress simulation performed at baseline; low stress shown in blue and high in red. Plaque burden at baseline (**D**) and follow-up (**F**); green indicates minimal thickness and red increased plaque thickness. There is significant plaque progression in the region of low shear stress at baseline (circle). Image courtesy of Dr Christos Bourantas.

Wall shear stress is typically estimated using computational fluid dynamics simulations that mimic coronary blood-flow based on reconstructed 3D geometry of the vessel. Anatomic information used for computational fluid dynamics simulation can be obtained with CCTA (Figure 5), or intravascular imaging combined with biplane angiography.^133^ Plaque structural stress is dependent on a variety of factors including arterial pressure, plaque composition and structure, tissue material properties, and luminal geometry. Plaque structural stress can be estimated using imaging data from VH-IVUS,^134^ and other methods, by using engineering techniques such as finite element analysis. Virtual computed FFR (CT_FFR_) can also be determined using biomechanical modeling, which shows good correlation to invasive FFR (*r*=0.7; *P*<0.001),^135^ with strong predictive power (area under the curve, 0.9) to detect CAD stenosis >50% when evaluated in clinical studies.^136^ CT_FFR_ can also potentially be used simulate hemodynamic changes resulting from stenting when planning invasive procedures^137^ and was shown to be useful as a method to streamline referrals for invasive angiography in a prospective longitudinal trial.^138^

### Risk Prediction and Biological Insights: What Have We Learned from Atherosclerosis Imaging Studies?

Fundamentally, the aim of atherosclerosis imaging is to identify patients most at risk of future clinical events, in an effort to best direct care and improve long-term outcomes. Imaging-based biomarkers for CVD provide important prognostic information, to compliment traditional clinical risk-stratification methods. These measurable imaging parameters are selected on their ability to identify (a) subclinical CVD in asymptomatic patients with risk factors, (b) obstructive or high-risk lesions in those with symptomatic CVD, or (c) high-risk patients with active CVD and extensive disease burden. Although tremendous resources over preceding decades have been devoted toward vulnerable plaque imaging, current evidence from prospective imaging studies fails to support this tactic, calling for a wider approach.^139^ Nevertheless, imaging atherosclerosis provides unparalleled insight into mechanisms underlying disease severity and progression, which continue to shape patient management. Here, we discuss the predictive value offered by each clinical imaging strategy, what has been learned about the natural history of atherosclerosis from important clinical studies, and the future role of multimodal imaging.

### Methods for CVD Screening

Limitations of clinical-risk scores drive search for cost-effective, noninvasive imaging tests, which can be rolled-out for screening individuals at risk of CVD. CAC scanning offers a simple, rapid, and reliable method of quantifying coronary calcium, which is pathognomic of established atherosclerosis. It is a powerful screening tool for asymptomatic patients with low to intermediate CVD risk, including those with diabetes mellitus, and can potentially improve adherence to lifestyle advice and medication.^11^ In the Multi-Ethnic Study of Atherosclerosis (MESA), which included 6722 patients followed up for median 3.8 years, increasing CAC conferred incremental rise in hazard ratio with a nearly 10-fold increased risk of a coronary event in those with CAC >300, and no difference among racial or ethnic groups.^140^ However, as dense calcium deposits seem to offer plaque stability,^141^ and progress with age and statin use,^142,143^ a high CAC score is perhaps most helpful as marker of disease burden, rather than for predicting likelihood of an event originating from an individual plaque. Importantly, for individuals with low and intermediate CVD risk, a CAC of zero is associated with <1% annual mortality over a 15-year period, irrespective of age or sex.^144^ As a result, absence of CAC reclassifies up to 50% of patients eligible for statins based on clinical risk scores alone.^145^ Carotid ultrasound is another important CVD screening tool; in the BioImage study (A Clinical Study of Burden of Atherosclerotic Disease in an At-Risk Population) of 5808 asymptomatic U.S. adults, 3-D assessment of carotid plaque burden using this inexpensive and portable technique appeared to provide similar risk prediction to coronary CAC scanning.^146^

### Imaging Biomarkers to Predict CVD Risk

The natural history of atherosclerosis is that of a slowly progressive, chronic disease; however, cardiovascular events arise suddenly and unexpectedly, with often devastating consequences for patients. In clinical practice, CAD is usually diagnosed on the basis of symptoms, plus the aid of anatomic or functional tests. Despite availability of numerous diagnostic tests, the timing and location of acute plaque disruption instigating clinical events cannot be reliably predicted by pre-existent anatomic stenosis severity, or indeed any other currently available method. This is because chronically obstructive plaques are less rupture-prone than TCFA owing to thick, heavily calcified fibrous caps,^39^ and only a minority of ruptured plaques result in clinical symptoms. More often, subclinical plaque rupture and healing occurs in the absence of multiple contributory factors promoting acute vessel occlusion.^147^ This highlights the need to consider additional factors beyond stenosis severity, including ischemic burden, plaque volume and vulnerability, microvascular and endothelial dysfunction, predilection to vasospasm, platelet function and coagulation, and current metabolic demands and inflammatory state.^148^

#### Stenosis Severity and Myocardial Ischemia

Current guidelines on coronary revascularization for symptomatic benefit in patients with stable CAD advocate a management strategy that incorporates functional assessment of myocardial ischemia in addition to angiography. Detection of ischemia helps to confirm obstructive lesions underlying angina symptoms, and is, itself, a strong prognostic marker. Indeed, long-term follow-up of the Fractional Flow Reserve Versus Angiography for Multivessel Evaluation (FAME) and Deferral versus Performance of Percutaneous Coronary Intervention of Functionally Nonsignificant Coronary Stenosis (DEFER) studies demonstrate the role of functional assessments to guide PCI for reducing stent use and need for repeat procedures.^27,149^ However, FFR-guided PCI does not seem to significantly lower rates of death or MI. Lack of improvement in hard-outcomes, such as MI or death, after PCI for stable angina versus medical therapy alone echoes previous findings of Clinical Outcomes Utilizing Revascularization and Aggressive Drug Evaluation (COURAGE),^150^ Bypass Angioplasty Revascularization Investigation 2 Diabetes (BARI-2D),^151^ and Medical, Angioplasty or Surgery Study II (MASS II) studies.^152^ This highlights potential inadequacies of a purely lesion-targeted approach and suggests an indirect relationship between individual stenosis severity and long-term prognosis.

It is clear that patients with multiple coronary stenosis are most at risk of future cardiac events, and that survival benefit is offered by coronary artery bypass surgery versus medical therapy±PCI in appropriately selected patients with multivessel CAD, particularly diabetics.^153^ For patients with left main stem, or 3 vessel CAD, angiographic anatomic complexity graded according to the SYNergy between PCI with TAXUS and Cardiac Surgery (SYNTAX) score is an important determinant of clinical outcomes when contemplating PCI versus coronary artery bypass surgery.^154^ The benefit of coronary artery bypass surgery over PCI for long-term survival might be because of the fact that a by-pass graft, as long as its patent, provides some protection against atherothrombotic events arising from any site proximal to the anastomosis, not just the original obstructive lesion.

When moving away from a plaque-centric model, stenosis severity remains nonetheless important as a marker of disease burden, and a degree of pre-existent luminal narrowing also seems necessary for acute thrombo-occlusion after plaque rupture.^155^ Major adverse clinical event prediction using FFR might also be improved among the most severe FFR values,^156^ and when combined with additional measures of microcirculatory hemodynamics.^31^ However, the link between myocardial ischemia and long-term prognosis remains elusive. Although, in theory, changes in local flow and stress because of hemodynamic obstruction could increase risk, the overall low MI rate in patients with stable angina,^3^ and apparent lack of survival benefit from ischemia-directed PCI suggest that myocardial ischemia might instead serve as a marker of advanced disease. The ongoing International Study of Comparative Health Effectiveness with Medical and Invasive Approaches (ISCHEMIA; NCT01471522) study aims to tackle this important question.

#### Plaque Burden and Vulnerability

Despite our ability to identify and characterize high-risk plaques, insights from prospective intravascular imaging trials question the significance of such findings. In Providing Regional Observations to Study Predictors of Events in the Coronary Tree (PROSPECT),^157^ VH-IVUS in Vulnerable Atherosclerosis (VIVA),^158^ and European Collaborative Project on Inflammation and Vascular Wall Remodeling in Atherosclerosis (ATHEROREMO-IVUS),^159^ the vast majority of TCFA identified with VH-IVUS did not result in subsequent MI or death over a period of 3 to 4 years. Although this important finding can, in part, be explained by the overall low positive predictive value of VH-IVUS to detect TCFA,^51^ there are several other potential contributory factors. First, we now know that there is a high incidence of subclinical plaque rupture at nonculprit coronary sites in patients with both ACS and stable CAD.^160^ We also know that transition to and from thin and thick-cap atheroma is relatively common within a 12-month period,^161^ and that cycles of rupture and healing can lead to variable rates of plaque progression.^162^ Indeed, for many patients with ACS rapid plaque progression occurs over a period of weeks to months,^163^ culminating in high burden of vulnerable disease and significant intraluminal stenosis acting as a nidus for thrombo-occlusive events.^164^

**Figure 6.**
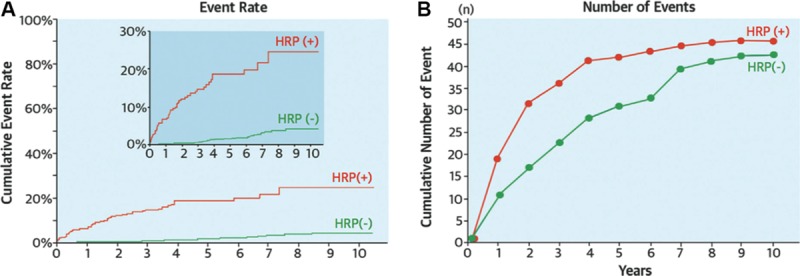
**Prediction of acute coronary syndrome (ACS) by high-risk computed tomographic (CT) features.** Results of prospective imaging study involving 3158 patients, aimed to evaluate whether CT-derived plaque characteristics can predict midterm likelihood of ACS. Cumulative event rate for patients with high-risk CT features (high-risk plaque [HRP] (+); low attenuation or positive remodeled plaque) identified at baseline vs those without high-risk CT features (HRP (−); **A**). Although the event rate in HRP (+) patients is higher than HRP (−), the number of patients in the HRP (+) group was 10-fold lower resulting in a similar cumulative number of events among the 2 groups (**B**). Adapted from Motoyama et al^167^ with permission of the publisher. Copyright ©2015, Elsevier.

Although most VH-IVUS defined TCFA do not become culprit lesions, high-risk plaque identification is nonetheless valuable as their presence portends future consequences of pancoronary vulnerability and disease progression.^165^ Furthermore, risk prediction can be improved by adopting an integrative approach that combines multiple markers of disease severity. In the Prediction of Progression of Coronary Artery Disease and Clinical Outcomes Using Vascular Profiling of Endothelial Shear Stress and Arterial Plaque Characteristics (PREDICTION) study, the combination of large plaque burden and low endothelial shear stress at baseline provided significant predictive value for identification of a lesion treated with PCI within 1 year.^166^ CCTA provides a good means to monitor plaque progression noninvasively. In a study of 3158 patients followed up for a mean 3.9 years after CCTA, both CT-defined high risk-plaque (positive remodeling or low attenuation) and plaque progression were important predictors of ACS, although the cumulative number of events was similar among patients with or without high-risk CT features at baseline (Figure 6).^167^ In the Prospective Multicenter Imaging Study for Evaluation of Chest Pain (PROMISE) study of roughly 10 000 symptomatic patients with intermediate pretest probability of CAD, a strategy of initial CCTA did not improve clinical outcomes when compared with functional testing over a median follow-up of 2 years.^168^ Use of semiautomated CT plaque segmentation tools,^56^ segmental calcium scoring,^169^ and coronary disease burden scores might further improve predictive accuracy of CCTA.^170^

#### Disease Activity

Atherosclerosis is driven by local and systemic inflammation, best imaged in vivo using ^18^F-FDG PET. In the Dublin Carotid Atherosclerosis Stroke Study, carotid artery inflammation detected by ^18^F-FDG was useful for identifying patients most at risk of early stroke recurrence, independent of age and stenosis severity.^91^ A retrospective study of 513 cancer-free patients also showed aortic ^18^F-FDG PET signal can predict CVD events independent of traditional risk factors (HR, 4.71; *P*<0.001), with nearly 30% net reclassification improvement over Framingham risk score in the highest-risk group and inverse relationship between PET signal intensity and timing of event.^92^ In a study of 1089 asymptomatic adults, both high carotid ^18^F-FDG uptake and carotid intima media thickness predicted stroke occurrence within 4 years; but only ^18^F-FDG provided additive value above Framingham risk score.^93^ After MI, increased ^18^F-FDG activity in the aorta also shows positive correlation to plasma troponin level.^171^ Unlike ^18^F-FDG, which has limited use in the coronaries, ^18^F-NaF localizes to individual coronary plaques with minimal background uptake and can reliably identify culprit coronary plaques post MI.^5^ Ongoing studies aim to evaluate the efficacy of prospective ^18^F-NaF coronary microcalcification imaging to identify high-risk prospective culprit lesions (NCT02110303 and NCT02278211).

Taking a wider view, imaging systemic networks of inflammation can potentially help clinical risk stratification and translational research. Using SPECT with ^99m^TC labeling of autologous peripheral blood monocytes, increased in vivo leukocyte trafficking was shown in relation to carotid plaque inflammation detected by ^18^F-FDG PET and atherosclerotic burden on MRI.^125^ The importance of measuring systemic inflammation was also demonstrated by a study that showed increased splenic metabolic activity after ACS to be an independent predictor of subsequent CVD events.^172^ Ectopic fat is increasingly recognized as another important CVD risk factor^173^; ^18^F-FDG uptake in tissue fat can be predicted by body weight and is strongly linked to arterial inflammation.^174^

### Multimodal Imaging Strategies

Given the highly complex, multifactorial pathophysiology of atherosclerosis and its progression to symptomatic plaque rupture, accurate risk prediction can potentially be improved in the future by multimodal imaging strategies that incorporate information from several complimentary imaging tools. This combined approach will be particularly relevant when assessing individual plaque vulnerability in patients with stable angina who might benefit from PCI. For example, PCI could be helpful to stabilize a high-risk lesion identified on basis of CT or VH-IVUS, if there is also evidence of hemodynamic obstruction, inflammation, or altered shear stress. With this aim, hybrid intravascular imaging catheters are being developed to combine, for example, detailed anatomic information gained from OCT imaging with simultaneous measurement of plaque composition using VH-IVUS or NIRS. Similarly, noninvasive hybrid PET/CT and PET/MR systems can in theory provide integrated measurement of factors including stenosis severity, markers of hemodynamic significance, myocardial viability, plaque burden and composition, and metabolic activity. Of course, not all information will be required for every case.

### Imaging for Drug Discovery and End Point for Clinical Trials

Research to identify and evaluate new atherosclerosis treatments is challenging. Despite best efforts only a handful of drugs have been proven to reduce atherosclerotic events. The ultimate step in drug evaluation (phase 3 trials) is to assess impact on clinical end points. Because of the nature of the disease, phase 3 atherosclerosis drug trials require enrolment of large numbers of patients (at times >20 000) and long follow-up periods, with typical costs exceeding $350 million.^175^ As a result of these enormous costs, it is important to gain clear insights of a drug’s potential for clinical efficacy during phase 2 trials. Atherosclerosis imaging allows measurable assessments of disease progression and activity, revealing early signals about potential drug effects, which can inform decisions and allow selection of drugs for phase 3 evaluation that have the most chance for success. In theory, imaging could potentially be used to select high-risk patients in effort to reduce the sample size needed in clinical outcome studies by increasing the outcome event rate; however, the practicality of this approach has yet to be determined. In certain circumstances, imaging data could also be used to help identify potential subgroups of patients in large trials who might benefit most from a particular treatment.

Both noninvasive and intravascular imaging modalities have been used to monitor effects of statins and other antiatherosclerosis drugs. Noninvasive methods are preferable for serial imaging in drug trials because of potential risks associated with invasive procedures. High participant drop out rates are also seen when invasive methods are used.^176^ However, much greater detail on plaque volume and composition can be gained with intravascular imaging, and radiation exposure is another major consideration when choosing between different imaging modalities. Change in plaque volume, composition, and inflammation has been used as imaging outcome measures during drug evaluation; however, clinical outcomes remain the only recognized route to drug approval. Luminal stenosis severity is less useful as this does not change much even with the most effective medical therapies, owing to the effect of positive vessel remodeling. Here, we provide a brief overview of some of the most informative atherosclerosis drug trials with imaging end points.

### Intravascular Imaging: IVUS and OCT

**Figure 7.**
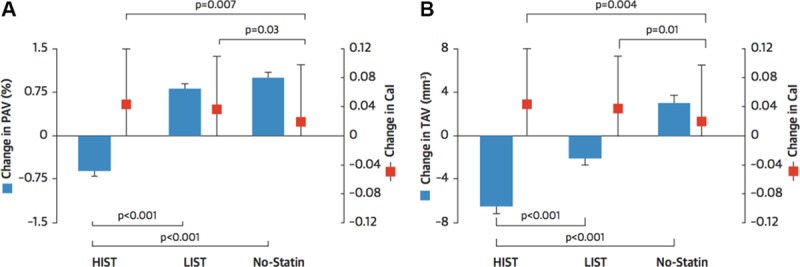
**Effects of statins on plaque morphology evaluated by intravascular imaging.** Summary of a post hoc analysis of 8 prospective randomized trials using serial intravascular ultrasound to detect change in percent atheroma volume (PAV; **A**), total atheroma volume (TAV; **B**), and calcium index (Cal) in response to low-intensity statin treatment (LIST) and high-intensity statin treatment (HIST). Significant plaque regression, with increased coronary atheroma calcification is observed in both low- and high-dose statin groups. Adapted from Puri et al^143^ with permission of the publisher. Copyright ©2015, Elsevier.

The beneficial actions of statins on CVD risk result from slowing disease progression and plaque stabilization, among other pleiotropic effects.^177^ Reduction in plaque volume after treatment with statins has been demonstrated by numerous clinical studies using intravascular imaging with IVUS and OCT, including A Study to Evaluate the Effect of Rosuvastatin on Intravascular Ultrasound-Derived Coronary Atheroma Burden (ASTEROID),^178^ Reversal of Atherosclerosis with Aggressive Lipid Lowering (REVERSAL),^179^ Early Statin Treatment in Patients With Acute Coronary Syndrome (ESTABLISH),^180^ and Integrated Biomarkers and Imaging Study-4 (IBIS-4).^181^ In 1 study, the combination of high-dose atorvastatin plus ezetimibe showed greater regression in plaque volume determined by IVUS than statins alone, mirroring changes in serum lipid levels.^182^ Significant reduction in plaque volume has also been shown following treatment with the angiotensin II receptor blocking agent olmesartan, when monitored with IVUS in 247 patients with stable angina over 14-month period.^183^ Studies using OCT to measure cap thickness have demonstrated a plaque stabilizing effect of statins, leading to increased cap thickness.^184^ Paradoxically, progression of plaque calcification is another potentially protective effect of statins shown by IVUS (Figure 7)^143,181^; however, change in coronary calcification has yet to undergo clinical evaluation as a marker of altered risk.^185^

### Noninvasive Imaging: Ultrasound, MRI, and CT

In patients with subclinical atherosclerosis, statins result in significant reduction in carotid intima media thickness when measured with ultrasound and MRI.^186,187^ Using 1.5-T carotid MRI, significant reduction in lipid-rich necrotic core content, but not plaque volume, was observed after treatment with rosuvastatin for 2 years in a randomized, double-blind phase 3 trial,^188^ and another in study after 1-year.^189^ Dalcetrapib, a cholesterol ester transfer protein (CETP) inhibitor that increases high-density lipoprotein, failed to reduce carotid artery wall thickness or volume measured by MRI^190^; a finding that presaged the dal-OUTCOMES trial, which showed that this drug also did not improve clinical outcomes.^191^ The National Institute on Aging (NIA) plaque study showed that the addition of niacin on top of statin therapy also did not reduce carotid wall volume,^192^ supporting clinical outcome data from the Atherothrombosis Intervention in Metabolic Syndrome with Low HDL/High Triglycerides: Impact on Global Health Outcomes (AIM-HIGH) study.^193^ In the Atorvastatin Therapy: Effects on Reduction of Macrophage Activity (ATHEROMA) study, reduction in carotid plaque inflammation in response to high-dose statin treatment was observed using USPIO MRI after just 3 months.^194^ Effects of the anti-inflammatory IL-1β monoclonal antibody canakinumab on carotid plaque burden will be quantified using MRI in a prespecified substudy of Cardiovascular Risk Reduction Study, Reduction in Recurrent Major CV Disease Events (CANTOS; NCT01327846). Although CCTA can potentially provide a useful noninvasive means of monitoring effects of drugs on coronary plaque volume and composition in noncalcified lesions,^195^ further evaluation of this application is needed, particularly in view of the added radiation risk.

### ^18^F-Fluorodeoxyglucose Positron Emission Tomography

Vascular inflammation imaging with ^18^F-FDG PET has been applied in several drug trials evaluating statins, and several other drugs, where anti-inflammatory effects (or lack thereof) were predictive of clinical efficacy. Significant reduction in plaque inflammation after treatment with statins has been demonstrated using ^18^F-FDG PET,^196^ with significant incremental dose–response^197^; consistent with known effects on low-density lipoprotein lowering and clinical outcomes. Reduction in arterial inflammation after treatment with pioglitazone, a diabetic drug with proven secondary preventive value, has also been observed.^198^ Conversely, failure to reduce arterial inflammation might suggest lack of clinical efficacy in atherosclerosis. Dalcetrapib, which is now known to be clinically ineffective, did not reduce arterial inflammation by any of the prespecified PET imaging measures in the dal-PLAQUE study.^190^ Similarly, a lipoprotein-associated phospholipase A2 inhibitor did not decrease atherosclerotic inflammation on PET imaging^199^ and failed to improve clinical outcomes in 2 randomized-controlled clinical trials.^200,201^

### Challenges of Noninvasive Coronary Imaging

Atherosclerosis imaging, particularly noninvasive methods, when focused on small and constantly moving coronary arteries, presents several hurdles. Technological advancements leading to reduction in radiation exposure and improved image quality, better spatial resolution, and more accurate motion correction are essential for the field to progress.

### Radiation Exposure

Most cardiovascular imaging techniques, including CT, SPECT, PET, and x-ray angiography, rely on radiation exposure to generate images of the heart and blood vessels. Significant radiation exposure is associated with an increased risk of cancer. Although the risk from a single scan is low, the effect of radiation exposure is cumulative over a life-time. Consequently, this restricts available methods for serial imaging and population screening; particularly as many patients will require additional scans for noncardiac reasons. Efforts to reduce exposure to ionizing radiation from medical imaging represent a major scientific endeavor. In particular, the advent of prospective cardiac gating and advances in CT scanner technology have dramatically reduced radiation exposure during CCTA. The average effective dose from CCTA with retrospective gating is 12 mSv; however, this is routinely reduced to ≈3.5 mSv with prospectively trigging. Diagnostic image quality is even possible at <1 mSv (comparable to chest x-ray exposure) using low tube-voltage (80–100 kV) and current (150–210 mA), along with iterative data reconstruction.^202^ Although MRI has a clear advantage, being radiation free, reliable imaging of the entire coronary vasculature remains elusive and hybrid PET/MR systems are not widely available.

### Spatial Resolution and Partial Volume Effects

The small caliber of coronary arteries, even in proximal segments (2–5 mm), demands high resolution anatomic images to differentiate specific plaque characteristics on CT and precisely localize PET signals. As a result, limited spatial resolution and partial volume effect can lead to problems such as calcium blooming artifact, resulting in overestimation of stenosis severity. Dual-energy CT is one plausible method to increase tissue contrast and allow better coronary plaque characterization^115^; however, this improvement seems to occur at the expense of reduced image quality compared with single energy scans in the clinical setting.^203^ Although coronary PET imaging is also problematic because of spill-in and spill-out of tracer activity, which can affect accurate signal quantification, this limitation can be partly corrected using a range of methods.^204^ Moreover, simultaneous motion correction and resolution recovery using PET/MR, coupled with advances in detector technology and signal processing methods, are likely to improve spatial resolution of PET.^205^

### Motion Artefacts

**Figure 8.**
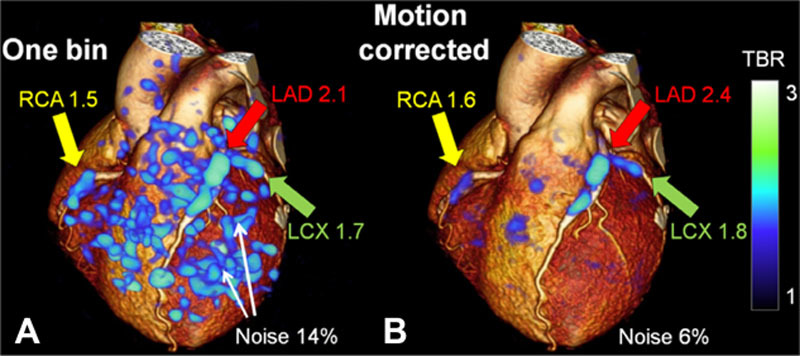
**Cardiac motion-corrected ^18^F-NaF positron emission tomography (PET)**. 3D cardiac computed tomographic (CT) rendering with superimposed ^18^F-NaF cardiac-gated PET image reconstruction using a single bin (25% of PET counts) vs (**B**) motion corrected PET with 10-gated bin method (consecutive 10% segments), resulting in less noise and improved target to background ratio. Adapted from Rubeaux et al^208^ with permission of the publisher. Copyright ©2016, Society of Nuclear Medicine and Molecular Imaging, Inc. LAD indicates left anterior descending artery; LCx, left circumflex artery; and RCA, right coronary artery.

The coronary arteries move with each heartbeat and respiratory cycle, creating complex motion patterns. X-ray angiography has high temporal resolution, allowing tracking of coronary movement in real time. Unlike invasive imaging, motion correction is a fundamental requirement for any noninvasive coronary imaging modality. Modern CT scanners are sufficiently fast to allow detailed single beat whole-heart imaging during a short breath hold using ECG-gating. PET and MR image acquisition takes much longer than CT, amplifying the negative impact of cardiac and respiratory movement on image quality. Although ECG-gating combined with breath hold acquisition, or respiratory motion tracking, is possible, these strategies are inefficient, as only a fraction of the data can be used. Self-navigation approaches introduced for MRI can track and correct for cardiac motion more effectively, leading to better scanning efficiency with the potential to reduce scanning time, motion blurring, and improve image quality.^206^ In principle, these methods could be applied to PET acquired simultaneously on hybrid PET/MR scanners, again improving efficiency and reducing radiation exposure. Alternatively, postprocessing methods for PET image motion correction include data-driven correction respiratory gating using deformation fields generated from 4D PET,^207^ and cardiac motion frozen technique.^208^ Motion correction of gated ^18^F-NaF coronary PET images is feasible and has been shown to reduce noise and increase tissue-to-background ratio compared with ungated and single-bin data (Figure 8).^208^ Such advances will, in future, help to facilitate translation of atherosclerosis imaging techniques on the horizon to routine clinical practice.

## Conclusions and Future Directions

In a new era of precision, personalized medicine, the diagnostic power of current and emerging atherosclerosis imaging methods will undoubtedly influence our approach to patient care and help drive cardiovascular research in effort to better understand disease mechanisms and test new treatments in the pipeline. With evolving technology and innovation, patient-specific multimodal imaging strategies can be tailored to reveal molecular signals with anatomic precision, while integrating data on plaque composition and local hemodynamics with markers of overall disease burden—thus, moving away from a plaque-centric approach, toward greater appreciation of the complexities underpinning the pathogenesis of atherosclerosis.

## Sources of Funding

J.M.T. is supported by a Wellcome Trust research training fellowship (104492/Z/14/Z). M.D. is supported by the British Heart Foundation (FS/14/78/31020). N.R.E. is supported by a research training fellowship from the Dunhill Medical Trust (RTF44/0114). A.J.B. is supported by the British Heart Foundation. J.H.F.R. is part-supported by the HEFCE, the NIHR Cambridge Biomedical Research Centre, the British Heart Foundation, and the Wellcome Trust.

## Disclosures

None.

## Supplementary Material
