# Supplementary material for: Imaging Atherosclerosis
Source: Circ Res. 2016 Feb 19;118(4):750–69. doi: 10.1161/CIRCRESAHA.115.306247 (PMC4756468; doi:10.1161/CIRCRESAHA.115.306247)
Supplement: Supplementary file 1 [file res-118-750-s001.doc]

*Circulation Research* Compendium on **Atherosclerosis**

Atherosclerosis: Successes, Surprises, and Future Challenges

Epidemiology of Atherosclerosis and the Potential to Reduce the Global Burden of Atherothrombotic Disease

Triglyceride-Rich Lipoproteins and Atherosclerotic Cardiovascular Disease: New Insights From Epidemiology, Genetics, and Biology

Genetics of Coronary Artery Disease

Surprises From Genetic Analyses of Lipid Risk Factors for Atherosclerosis

From Loci to Biology: Functional Genomics of Genome-Wide Association for Coronary Disease

Are Genetic Tests for Atherosclerosis Ready for Routine Clinical Use?

Endothelial Cell Dysfunction and the Pathobiology of Atherosclerosis

Macrophages and Dendritic Cells - Partners in Atherogenesis

Macrophage Phenotype and Function in Different Stages of Atherosclerosis

Adaptive Response of T and B Cells in Atherosclerosis

Microdomains, Inflammation and Atherosclerosis

Vascular Smooth Muscle Cells in Atherosclerosis

MicroRNA Regulation of Atherosclerosis

The Success Story of LDL Cholesterol Lowering

From Lipids to Inflammation – New Approaches to Reducing Atherosclerotic Risk

Imaging Atherosclerosis

***Guest Editors: Peter Libby, Karen Bornfeldt, Alan Tall***

[Note: This is the master text box for this series. For each article this text box appears in, please make the title above of that article bold.]
