# Supplementary material for: Imaging Atherosclerosis
Source: Circ Res. 2016 Feb 19;118(4):750–69. doi: 10.1161/CIRCRESAHA.115.306247 (PMC4756468; doi:10.1161/CIRCRESAHA.115.306247)
Supplement: Supplementary file 3 [file res-118-750-s003.pdf]

Wolters Kluwer

**Title:**	Identification of Fibrous Cap Rupture With Magnetic Resonance Imaging Is Highly Associated With Recent Transient Ischemic Attack or Stroke
**Author:**	Chun Yuan, Shao-xiong Zhang, Nayak L. Polissar, Denise Echelard, Geraldo Ortiz, Joseph W. Davis, Elizabeth Ellington, Marina S. Ferguson, Thomas S. Hatsukami
**Publication:**	Circulation
**Publisher:**	Wolters Kluwer Health, Inc.
**Date:**	Jan 15, 2002

Copyright © 2002, Wolters Kluwer Health

Logged in as:
Jason Tarkin
Account #:
3000631111
LOGOUT

Review Order

Please review the order details and the associated terms and conditions.

Licensed Content Publisher	Wolters Kluwer Health, Inc.
Licensed Content Publication	Circulation
Licensed Content Title	Identification of Fibrous Cap Rupture With Magnetic Resonance Imaging Is Highly Associated With Recent Transient Ischemic Attack or Stroke
Licensed Content Author	Chun Yuan, Shao-xiong Zhang, Nayak L. Polissar, Denise Echelard, Geraldo Ortiz, Joseph W. Davis, Elizabeth Ellington, Marina S. Ferguson, Thomas S. Hatsukami
Licensed Content Date	Jan 15, 2002
Licensed Content Volume	105
Licensed Content Issue	2
Type of Use	Journal/Magazine
Requestor type	Academic/Educational
Format	Print and electronic
Portion	Figures/table/illustration
Number of figures/tables/illustrations	1
Figures/tables/illustrations used	Figure 2
Author of this Wolters Kluwer article	No
Will you be translating?	No
Title of new article	Imaging Atherosclerosis
Publication the new article is in	Circulation Research
Publisher of new article	Wolters Kluwer Health, LWW
Author of new article	Jason M. Tarkin, Marc R. Dweck, Nicholas R. Evans, Richard A.P. Takx, Adam J. Brown Ahmed Tawakol, Zahi A. Fayad, James H.F. Rudd
Expected publication date of new article	Jan 2016
Estimated size of new article (pages)	10
Requestor Location	Jason M Tarkin<br>Division of Cardiovascular Medicine, University of Cambridge<br>Box 110, ACCI, Addenbrooke's Hospital<br>Hills Road<br>Cambridge, United Kingdom CB2 2QQ<br>Attn: Jason M Tarkin
Total	115.00 USD

Edit Order Details

Edit Requestor Location   This location may be used to determine your tax liability

☐ I agree to these terms and conditions.

☐ I understand this license is for reuse only and that no content is provided.

Customer Code (if supplied)   APPLY

BACK   DECLINE   CHOOSE PAYMENT

Copyright © 2015 Copyright Clearance Center, Inc. All Rights Reserved. Privacy statement. Terms and Conditions.
Comments? We would like to hear from you. E-mail us at customercare@copyright.com
