# Supplementary material for: Imaging Atherosclerosis
Source: Circ Res. 2016 Feb 19;118(4):750–69. doi: 10.1161/CIRCRESAHA.115.306247 (PMC4756468; doi:10.1161/CIRCRESAHA.115.306247)
Supplement: Supplementary file 7 [file res-118-750-s007.pdf]

Subject	**RE: Reprint Permission Request Form Form Submission**
From	Permissions <permissions@snm.org>
Sender	Sumimoto Mark <msumimoto@snmmi.org>
To	jt545@cam.ac.uk <jt545@cam.ac.uk>
Date	2015-12-10 23:13

Dear Jason Tarkin,

JNM has an open permission policy allowing the use of original figures and tables published in JNM in other journals. In these cases, formal permission is not required as long as proper credit is given to JNM as follows: This research was originally published in JNM. Author(s). Title. J Nucl Med. Year;vol:pp-pp. © by the Society of Nuclear Medicine and Molecular Imaging, Inc.
This policy is posted online at: http://jnm.snmjournals.org/site/misc/permission.xhtml. (The final article with complete citation information will be published at the beginning of January 2016.)

Materials credited to outside sources are not original. In this case, the original rightsholders should be contacted for reprint permission. Copyright to material published by presenters at SNMMI Annual Meetings is retained by the original authors.

To reprint JNM material in other types of publications, please complete the online reprint permission form at: http://www.snmmi.org/Applications/Forms/FormDisplay.aspx?FormID=23215&navItemNumber=684.

Mark Sumimoto
SNMMI | SOCIETY OF NUCLEAR MEDICINE AND MOLECULAR IMAGING
Editorial Project Manager
SNMMI | The Society of Nuclear Medicine and Molecular Imaging
phone: 703.652.6777 | fax: 703.708.9018
email: msumimoto@snmmi.org
www.snmmi.org

Get instant access to 100 of the most popular sessions from SNMMI's 2015 Annual Meeting with the SNMMI Virtual Meeting.

From: automail@cms-plus.com [mailto:automail@cms-plus.com]
Sent: Saturday, December 05, 2015 6:19 AM
To: Permissions <permissions@snm.org>
Subject: Reprint Permission Request Form Form Submission

The following was submitted on www.snmmi.org as ItemNumber 74272 of form *Reprint Permission Request Form*

Requestor Information:

Full Name:	Jason Tarkin
Organization:	University of Cambridge
Address 1:	Box 110, ACCI, Addenbrooke's Hospital

Address 2:	Hills Road
City:	Cambridge
State/Province:	CAMBS
ZIP/Postal Code:	CB2 0QQ
Phone:	+44(0)1223331504
Fax:	+44(0)1223331505
Email:	jt545@cam.ac.uk

Tell Us About Your Publication:

Authors/Editors:	Jason M. Tarkin, Marc R. Dweck, Nicholas R. Evans, Adam J. Brown, Ahmed Tawakol, Zahi A. Fayad, James H.F. Rudd
Title of Publication:	Atherosclerosis Compendium
Article Title (if applicable):	Imaging Atherosclerosis
Projected Date of Publication:	Early 2016
Publisher:	Circulation Research
Place of Publication:	Journal, online
Purpose of Publication (Educ., Comm., Other)	Educational

What SNMMI Publication Material Would You Like To Reprint?

Authors:	Mathieu Rubeaux, Nikhil Joshi, Marc R Dweck, Alison Fletcher, Manish Motwani, Louise E Thomson, Guido Germano, Damini Dey, Debiao Li, Daniel S. Berman, David E Newby, and Piotr J Slomka
Title of Publication:	Motion correction of 18F-sodium fluoride PET for imaging coronary atherosclerotic plaques
Article Title:	
Year:	2015
Volume:	
Issue Number (if reprinting from a journal):	
Page Numbers of Article:	1-26
Figure or Table Number(s):	Figure 5
Page Numbers of Material in Question:	24
Additional Information:	
