# Supplementary material for: Imaging Atherosclerosis
Source: Circ Res. 2016 Feb 19;118(4):750–69. doi: 10.1161/CIRCRESAHA.115.306247 (PMC4756468; doi:10.1161/CIRCRESAHA.115.306247)
Supplement: Supplementary file 8 [file res-118-750-s008.pdf]

Copyright Clearance Center RightsLink®

Creative Commons Attribution License (CC BY)

This article is available under the terms of the Creative Commons Attribution License (CC BY). You may distribute and copy the article, create extracts, abstracts, and other revised versions, adaptations or derivative works of or from an article (such as a translation), to include in a collective work (such as an anthology), to text or data mine the article, including for commercial purposes without permission from Elsevier. The original work must always be appropriately credited.

Permission is not required for this type of reuse.

CLOSE WINDOW

Copyright © 2015 Copyright Clearance Center, Inc. All Rights Reserved.
Comments? We would like to hear from you. E-mail us at customercare@copyright.com
